# Resources for innovative learning of anatomy and foot ossification: Graphic design and virtual reality

**DOI:** 10.1002/jfa2.70008

**Published:** 2024-09-28

**Authors:** Ana Cristina Moreno‐Marin, Manuel Pardo Rios, Eva Lopezosa‐Reca, Cristina Molina García, Salvador Díaz‐Miguel, Beatriz Gómez‐Martín, Paula Cobos‐Moreno

**Affiliations:** ^1^ Grupo de Investigación de Nuevas Tecnologías para la Salud Universidad Católica de Murcia (UCAM) Guadalupe, Murcia Spain; ^2^ Department Nursing and Podiatry University of Malaga (UMA) Málaga Spain; ^3^ Nursing Department University of Extremadura (UEX) Extremadura Spain

**Keywords:** anatomy, bone growth, foot, ossification, virtual anatomical model, virtual reality

## Abstract

**Introduction:**

This study addresses the ossification process of the foot, a topic of great relevance within podiatry courses. Understanding the chronology of foot bone formation is essential for evaluating pathological processes and establishing appropriate therapeutic actions to improve patient quality of life. The main objectives of this work are to understand the ossification process of the foot bones and to propose an appropriate didactic methodology for effective learning of this process.

**Materials and Methods:**

The individual ossification sequences of the foot bones were established and virtually recreated to make these processes more didactic and usable as teaching aids. The literature search was conducted using the PRISMA statement, focusing on terms, such as “bone ossification,” “foot,” and “bone development,” and included relevant studies from medical databases.

**Results:**

Updating the ossification ages and providing previously unavailable visual teaching material offers a useful tool for improving the teaching of this subject. It was found that, in general, the tarsal bones show significant differences in ossification ages between sexes, with later and slower ossification in males. These differences are statistically analyzed and presented in detailed comparative tables.

**Conclusions:**

The use of innovative teaching tools, such as virtual anatomical models, helps students to better understand the ossification process of foot bones. Implementing these tools in the podiatry curriculum not only facilitates knowledge acquisition but also enhances the quality of teaching and, consequently, the future clinical practice of students.

## 1 INTRODUCTION

Higher education has undergone numerous changes in recent years. The widespread use of virtual education and information and communication technologies (ICTs) means that education today is very different from the classical trends of a few years ago [1].

Motivated by the consequences of a global pandemic and the resulting lockdown, current educators have had to adapt their teaching methods [2]. Regularly, this even involves modifying the dynamics of student training, including the possibility of remote education, thus eliminating distance and time barriers [3]. The use of virtual tools, new technologies, and specialized software offers suitable alternatives to improve teaching [4].

In recent years, the use of multimedia tools has been implemented in podiatry education [5], and we even have studies of the impact of the introduction of a multimedia learning tool in podiatric medicine courses [6]. This study is the first to develop specific virtual anatomical models for foot ossification, providing an innovative educational tool for podiatry students.

In the teaching process, where the educator seeks the acquisition of knowledge by students, two parts must be distinguished. While the curriculum model defines the content to be covered, the didactic methodology specifies the actions, processes, and strategies that can be carried out to transmit knowledge [7]. In our case, we use the curricular context to independently teach the ossification process of the foot bones. This subject is traditionally included in several mandatory podiatry courses such as anatomy, pathology, and pediatrics. It is crucial for students in these disciplines to thoroughly understand this process, as a specific diagnosis and consequently a successful treatment that improves the patient's quality of life depend on it.

However, the scarcity of recent publications that unify the ossification times of foot bones and the density of concepts to be transmitted make studying and understanding this subject very difficult for students. New didactic methodologies help students deeply understand the concepts presented in the classroom [8]. The use of active methodologies allows students to be the protagonists of their own learning process, simultaneously acquiring skills and knowledge [9]. ICTs integrate into active methodologies as tools that allow access to information and collaboration, offering new educational scenarios and expanding beyond the classroom boundaries [10].

In this context and given a challenging curricular context, the use of graphic and virtual design tools allows the creation of animated and unprecedented anatomical models that allow a better understanding of the subject to be taught. Thus, the objectives of this work are, on the one hand, to determine the individualized ossification process of the bones of the foot and, on the other hand, to use this information to design virtual anatomical models that improve the understanding of the ossification process.

## 2 METHOD

To digitally create animated virtual anatomical models that simulate the real process of ossification of the bones of the foot, the Blender software was used, which allows the creation of 3D models, and the Unity software, for the development of the virtual reality environments. It is of vital importance to define this process individually for each of the bones that make up the skeleton of the foot. To do this, a bibliographic search was carried out to determine the current state of scientific knowledge on the chosen study topic. To adapt the methodology of this work to the established criteria, the PRISMA declaration (Preferred Reporting Items for Systematic Reviews and Meta‐Analyses) was considered [11].

**FIGURE 1:**
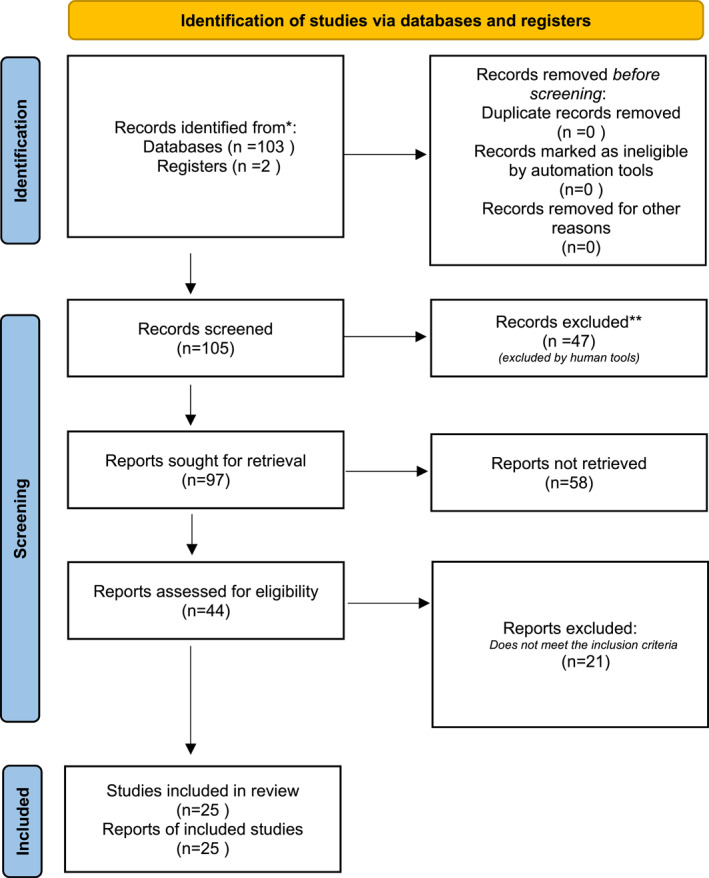
PRISMA 2020 flow diagram for new systematic reviews. *Consider, if feasible to do so, reporting the number of records identified from each database or register searched (rather than the total number across all databases/registers). **If automation tools were used, indicate how many records were excluded by a human and how many were excluded by automation tools.

The results of the systematic search are summarized in the flow diagram (Figure 1). The search terms used were “bone ossification,” “foot,” and “bone development” between December 2023 and March 2024 in medical databases such as PubMed, Elsevier, and SciELO.

*Inclusion criteria*:

- Content related to the ossification process of human foot bones.

*Exclusion criteria:*

- Growth anomalies.
- Surgical interventions/abnormal evolution.

## 3 RESULTS

After an exhaustive search, a total of 25 articles were selected after applying the inclusion and exclusion criteria. The literature review identifies that the existing evidence is very scarce, outdated, and none of them proposed empirical conclusions on the ossification process of foot bones. After a detailed analysis of the ossification ages established by classical authors, a comparison is made with the ossification ages of foot bones established in the only recent empirical work with gender differentiation [12].

Therefore, and to provide an analytical update to the temporal sequence in foot bones, the results obtained from the only recent scientific study with descriptive results of the ossification process of the human foot in pediatric populations are analyzed [12].

The data contrast study between ossification ages in boys and girls is performed using the paired samples *t*‐test and Levene's test for homogeneity of variances to verify the hypothesis of variance equality between sexes. The bone age is converted to months to enable better statistical treatment of the data.

All the bones forming the tarsus show statistical significance in the sex comparison, except for the cuboid, navicular, cuneiforms, and metatarsals. The rest of the tarsus bones show a significant difference (*T* ≤ 0.05; *t* test), translating into a later and slower ossification rate for males. For better understanding, the comparative table (Table 1) summarizes the ossification ages of the foot bones (expressed in months) based on the established literature.

**TABLE 1:** Comparison of ossification ages of foot bones according to existing literature.

Bones	Ossification (months) systematic review		Ossification (months) Cobos‐Moreno 2023		*p*‐value
	Male	Female	Male	Female	
Calcaneus	132–168 (Hoerr et al. [13])	120–144 (Hoerr et al. [13])	189	143	0.045
Talus	144 (Hoerr et al. [13]; O'Rahilly & Gardner [14]; Tachdjian [15]; Wakely et al. [16])	108 (Hoerr et al. [13]; O'Rahilly & Gardner [14]; Tachdjian [15]; Wakely [16])	156	156	0.004
Cuboid	96 (Scheuer & Black [17])	96 (Scheuer & Black [17])	156	156	0.003
Navicular	96 (Becerro de Bengoa et al. [18])	96 (Becerro de Bengoa et al. [18])	156	156	0.004
Lateral cuneiform	144 (Krogman & Isçan 1986 [19]; Scheuer & Black [17])		156		0.046
Medial cuneiform					
Intermedial cuneiform					
Metatarsal	168–204 (Scheuer & Black 2004 [17])		192		0.052
Phalanges	168–192 (Scheuer & Black 2004 [17])		172	192	0.055
Sesamoid bones	108 (Scheuer & Black 2004 [17])		108	96	0.034

## 4 DISCUSSION

Overall, the results obtained from the complex study of bone maturation in foot bones largely coincide with the proposals of all authors. However, the minor variations and detailed study are small details to consider for enriching the acquisition of knowledge on this topic and striving for excellence, both in teaching and clinical practice.

It should be noted that there are certain situations where the accuracy of age estimation is extremely important; clinical diagnoses of growth disorders, legal implications, estimation of the age of skeletal remains, or judicial processes, among others. For this reason, it is considered of great interest to refine the age estimation of an individual as much as possible. Additionally, a simplified teaching approach should be considered to help students understand this complex process. Therefore, in the future, bone maturation ages will be referred to in years, making the corresponding calculation based on the ages established and presented in the previous section (in months).

**FIGURE 2:**
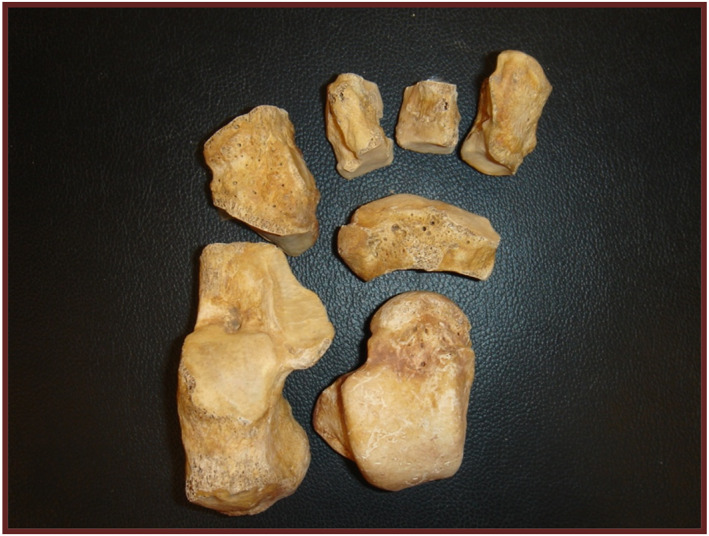
Tarsal bones. From right to left and from bottom to top: astragalus, calcaneus, scaphoid, cuboid, medial cuneiform, intermediate cuneiform, and lateral cuneiform.

For a better understanding of the bone maturation process of the foot, it is advisable to divide the foot skeleton into three easily identifiable areas: rearfoot, midfoot, and forefoot [20]. The rearfoot and midfoot consist of a set of seven bones: the talus (os talus), the calcaneus (os calcaneus), the navicular (os naviculare pedis), the cuboid (os cuboides), and the three cuneiforms (ossa cuneiformia) (Figure 2).

Their ossification is characterized by belonging to the second group of the primary ossification centers in the foot. They appear chronologically after the metatarsals and phalanges, which constitute the first group. Although most of them appear after birth, they all have exponential growth in the early years of life. The first ossification center to appear is the calcaneus, followed by the talus and the cuboid. The rest of the tarsal bones always appear after birth, following a temporal sequence that begins with the third cuneiform or lateral cuneiform, the first cuneiform or medial cuneiform, and the intermediate cuneiform or second cuneiform. The navicular is the last bone to appear and begin ossification.

The calcaneus is the longest bone of the tarsus bones and constitutes the anatomical region known as the heel. It articulates superiorly and anteriorly with the talus and distally with the cuboid. It is the first tarsal bone to begin ossification, starting from two separate and independent ossification centers (primary and secondary) (Table 2).

**TABLE 2:** Morphological index of the calcaneus.

Period	Event	Source
Fetal (age)		
4–6 months	Appearance of the primary ossification center	O'Rahilly and Gardner [21], Meyer and O'Rahilly [22]
Postnatal (age)		
4 years girls	Early appearance of the second ossification center	Birkner [23], Tachdjian [15]
5–6 years girls	Normal appearance of the second ossification center	Scheuer and Black [17]
7–8 years boys		
10 years boys	Late appearance of the second ossification center	Birkner [23], Tachdjian [15]
10–12 years girls	Complete epiphyseal fusion	Hoerr et al. [13]
11–14 years boys		

The primary ossification center is present at birth and initially appears as a rounded ossicle. Over time, it morphologically differentiates to acquire its definitive adult form. Sequentially, the anterior face of the bone begins to flatten, forming the facet that will articulate with the cuboid. Subsequently, the facets that will articulate with the talus become evident and the calcaneus generally grows in length. The bone grows exponentially and undergoes constant changes until the age of two. After that, its growth slows until puberty, where changes are gradual and small.

The second ossification center of the calcaneus is considered a traction epiphysis associated with the attachment of the Achilles tendon. Its appearance can occur as a single or polyfragmented ossicle from ages 5–6 in girls and 7–8 in boys [12], although some authors claim it can appear very early in girls (4 years) [17] and late in boys (10 years) [13]. Whether single or multiple, the second ossification center typically appears below the middle of the posterior edge of the calcaneus, extending longitudinally with growth, both distally and proximally, until it completely covers the posterior part of the calcaneus such as a cap around the age of 8 in girls and 10 in boys [20]. The epiphyseal union occurs around ages 10–12 in girls and 11–14 in boys [12, 13].

This complex process of bone formation is depicted in the virtual recreation available at the following link (ossification of the Calcaneus) The sequential recreation of the entire process is considered useful from a didactic point of view as it facilitates understanding and aids in studying.

The talus is the tarsal bone that articulates with the tibia and fibula at its distal end, forming the ankle joint. Its direct articular relationship with the calcaneus is also noteworthy, forming the subtalar joint, which is crucial for proper foot movement during ambulation.

The talus ossifies from two ossification centers (Table 3). The primary ossification center is present at birth, rounded and completely undifferentiated. After birth, it begins to differentiate, leading to the formation of the trochlea (which will articulate with the tibia and fibula to form the ankle), the anatomical neck, and the lateral process (which will eventually form the sinus tarsi region) [20].

**TABLE 3:** Morphological index of the talus.

Period	Event	Source
Fetal (age)		
6 months girls	Appearance of the primary ossification center	Fazekas and Kósa [24], Stripp and Reynolds [25]
7 months boys		
Postnatal (age)		
8 years girls	Appearance of the second ossification center (*os trigonum*)	Turner [26], Wakely et al. [16]
11 years boys		
9 years girls	Complete epiphyseal fusion (if occurring)	O'Rahilly and Gardner [14], Hoerr et al. [13], Tachdjian [15], Wakely et al. [16]
12 years boys		

The second ossification center of the talus, also known as the posterior epiphysis, does not always appear. When it does, two situations can occur. The first is that it fuses with the primary ossification center or body of the bone, forming what is anatomically called the talus tail. The second is that it evolves as an independent ossicle and never fuses with the primary ossification center. In this case, it forms an accessory bone called the os trigonum, present in approximately 5% of the general population [13, 14].

When the second ossification center is present, regardless of whether it fuses with the talus body, it appears around 8 years in girls and 11 years in boys [12, 15]. If it fuses with the primary center, the fusion occurs within a year of its appearance [16, 20]. This maturation process is depicted in the educational video available at the following link (ossification of the Talus).

The chronological process of the bones forming the midfoot begins with the appearance of the cuboid (Table 4). The cuboid's ossification often starts before birth (36–40 weeks of fetal life) [14]. It is not uncommon for it to appear after birth (around 3 months in girls and 6 months in boys) (Scheuer & Black [17]).The presence of the cuboid at birth is useful for some authors, along with the study of the proximal tibial epiphysis, to establish bone maturation in the perinatal period in cases with clear medico‐legal implications [19]. However, other authors argue that the presence or absence of the cuboid ossification center is not a reliable indicator in a full‐term fetus [29].

**TABLE 4:** Morphological index of the cuboid.

Period	Event	Source
Fetal (age)		
36–40 weeks	Appearance of the primary ossification center	Scheuer and Black [17]
Postnatal (age)		
3 months girls	Appearance of the primary ossification center (sometimes)	Pyle and Sontag [27], O'Rahilly and Gardner [14], Hoerr et al. [13], Acheson [28]
6 months boys		
8 years	End of ossification (adult‐like form)	Scheuer and Black [17]

Between 6 months and 1 year after birth, the medial surface (which will later articulate with the lateral cuneiform) begins to flatten. Between 3 and 4 years of life, the ossicle starts to differentiate, losing its perfectly rounded shape and forming the characteristic corners of its final morphology. By 4 years, the articular facets are completely defined. By 8 years, the bone has the same shape as an adult bone but is smaller in size [12]. The maturation process is depicted in the educational video available at the following link (ossification of the cuboid).

The lateral cuneiform appears subsequently as a single rounded ossicle between the sixth month and the first year of life [17]. At the end of the first year, it begins to lose its rounded shape, differentiating the articular facets that will be clearly visible around 4 years. Between 4 and 6 years, the bone has acquired a morphology similar to that of an adult bone [12].

The medial cuneiform or the first cuneiform is the second to appear among the three cuneiforms. It begins ossification in the second year of life in girls and the third year in boys. Around the age of 6, it usually completes its morphological differentiation, acquiring the adult form [29].

The intermediate cuneiform, also called the second cuneiform, appears last among the three and is the sixth bone to appear among the tarsal bones. It appears as a single ossification center at 2.5 years in girls and 3.5 years in boys, although some authors claim that it may appear later in boys up to 4 or even 5 years (Table 5). Around 6 years, it usually completes its morphological differentiation, such as the other cuneiforms, acquiring the adult form. However, the three ossicles will not complete their maturation process until 11–12 years [12, 17, 20]. The chronological and bone growth process of the three cuneiforms is depicted in the educational video available at the following link (ossification of cuneiform).

**TABLE 5:** Morphological index of the cuneiforms.

Period (postnatal)	Event	Source
Fetal (age)		
Lateral cuneiform		
3–4 months	Appearance of the primary ossification center	Elgenmark [30]
5–6 months		
4–6 years	Adult bone	Scheuer and Black [17]
Medial cuneiform		
2 years and 11 months	Appearance of the primary ossification center	Elgenmark [30]
4 years and 3 months		
6 years	Adult bone	Scheuer and Black [17]
3–4 years	Os cuneiforme (sometimes)	O'Rahilly and Gardner [14], Acheson [28]
Intermedial cuneiform		
2 years and 11 months	Appearance of the primary ossification center	Elgenmark [30]
4 years and 3 months		
6 years	Adult bone	Scheuer and Black [17]

The navicular, also called the scaphoid, is the last tarsal bone to begin ossification (Table 6) and usually does not appear before 2.5 years in girls and the beginning of the fourth year in boys [13]. However, there is a significant variation among the multiple publications on this topic, with the appearance period ranging from 2 to 6 years [18]. Some authors conclude that the navicular ossification center should always be present in children older than 4 years and 3 months. Around the age of 5, the bone acquires a vaulted shape both distally and proximally. When the individual reaches 7–8 years, the bone has defined its adult‐like form [17]. The second ossification center of the navicular is not constant, appearing around 9 years in girls and 12 years in boys. In most cases, the new ossicle fuses with the primary ossification center, forming a single bone in adulthood [20]. The maturation age is established between 10 and 12 years [12]. The maturation process is depicted in the educational video available at the following link (ossification of the Navicular).

**TABLE 6:** Morphological index of the navicular.

Period	Event	Source
Post natal (age)		
2 and 5 years girls	Appearance of the primary ossification center	Frazer [31], Hoerr et al. [13], Acheson [28], Stripp and Reynolds [25]
4 years boys		
7–8 years	Complete differentiation (adult‐like form)	Scheuer and Black [17]
9 years girls	Appearance of the second ossification center (not constant) (accessory navicular)	Becerro de Bengoa et al. [18]
12 years boys		

**FIGURE 3:**
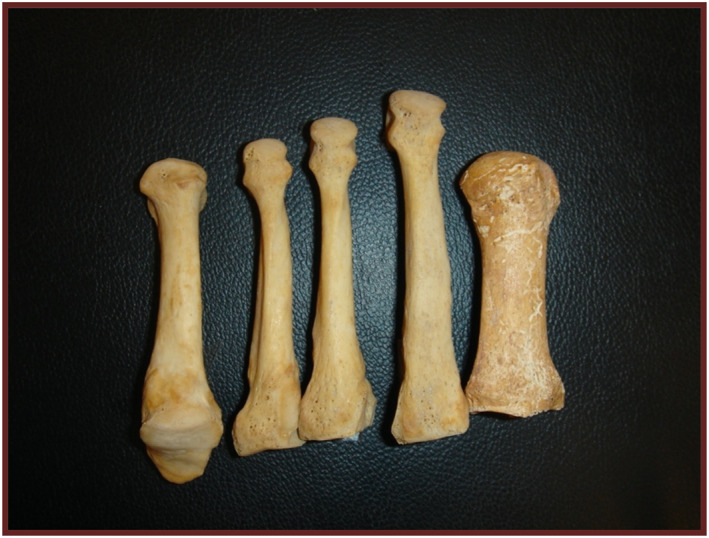
Metatarsal bones. From right to left: first metatarsal, central metatarsal, and fifth metatarsal.

The forefoot consists of a set of bones including the metatarsals and phalanges. The metatarsals are five long bones located distal to the tarsal bones and proximal to the phalanges, contributing to the foot's architecture (Figure 3).

The primary ossification centers of the metatarsals are already present at birth as single nuclei that will differentiate into their diaphyses (Table 7). They appear around 8–10 weeks of intrauterine life with an ossification sequence similar to the metacarpals of the hand. The second, third, and fourth metatarsals appear first, followed by the fifth. The first metatarsal is the latest to appear, around the 12th week of fetal life [17].

**TABLE 7:** Morphological index of the metatarsals.

Period	Event	Source
Natal (age)		
8–10 weeks	Appearance of the primary ossification center (2nd–5th metatarsals)	Frazer [31], Jit [32], Hoerr et al. [13], Birkner [23], Fazekas and Kósa [24]
12 weeks	Appearance of the primary ossification center (1st metatarsal)	
Postnatal (age)		
2–3 años	Appearance of the secondary ossification center distal (2nd–5th metatarsals)	Franch et al. [33]
2 años	Appearance of the secondary ossification center proximal (1st metatarsal)	
14–17 years	Epiphyseal union	Scheuer and Black [17]

The secondary ossification centers vary, as the first metatarsal appears at its proximal base as a flattened single ossicle (ossifying such as a phalanx). The rest appear as rounded nodules that will differentiate into the metatarsal heads. In any case, their appearance is evident but undifferentiated, being earlier in the first metatarsal (around 2 years) followed by the rest between 2 and 3 years [33]. They remain as undifferentiated ossicles until 4–5 years. Their epiphyseal fusion begins at 14 years, extending until adulthood, approximately 17 years [12]. The chronological and bone growth process of the metatarsal bones is depicted in the educational video available at the following link (ossification of the metatarsal bones of the foot).

**FIGURE 4:**
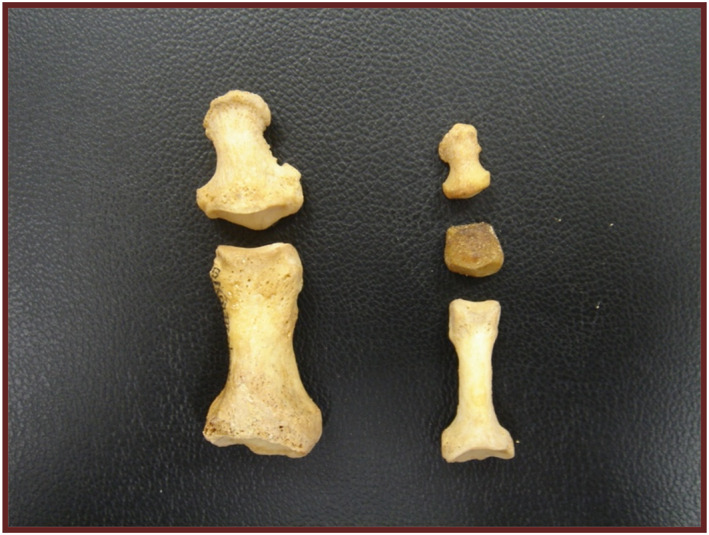
Bone structure of the toes. On the left, the proximal and distal phalanges of the hallux and on the right, the proximal, middle, and distal phalanx as an example of any toe of the foot.

Each toe consists of three phalanges: proximal, middle, and distal, except for the first toe or hallux, which has only two: proximal and distal (Figure 4).

The phalanges generally ossify from two ossification centers, one is the primary that differentiates into the diaphysis or body of the phalanx and the other is the secondary that forms the bases (Table 8). The distal phalanges show great variability in the appearance of their first ossification center. The distal phalanx of the first toe appears in the seventh week of intrauterine life, followed by the second, third, and fourth toes around the ninth week of fetal life. The distal phalanx of the fifth toe appears later, around the 11th or 12th week, possibly delaying its appearance until the fifth or sixth month of life [14].

**TABLE 8:** Morphological index of the phalanges.

Period	Event		Source
Natal (age)			
7 weeks	Appearance of the primary ossification center	1st toe	O'Rahilly and Gardner [14]
9 weeks		2nd–4th toe	Birkner [23]
11–12 weeks		5th toe	Scheuer and Black [17]
Postnatal (age)			
9 months girls	Appearance of the secondary ossification center	1st toe	Scheuer and Black [17]
14 months boys			
2–3 years girls		2nd–5th toe	
4–5 years boys			
11–13 years girls	Epiphyseal union	1st–5th toe	
14–16 years boys			

The second ossification center in the first toe does not appear until 9 months of life in girls and 14 months in boys. In the other toes, the second ossification center of the distal phalanx does not appear until 2–3 years in girls and 4–5 years in boys. Their epiphyseal fusion occurs after a long process of bone differentiation around 11–13 years in girls and 14–16 years in boys [12, 17]. The maturation process of the metatarsals and phalanges is depicted in the educational video available at the following link (ossification of the phalanges of the foot).

These differences suggest that the diagnosis and treatment of foot pathologies should consider sex‐specific variations in ossification. The development of videos showing the process of foot growth as a tool for higher education is a pilot project and is intended to be implemented in the future. Despite the antecedents present in the scientific literature, we have to think that the results are going to be encouraging, as some authors already show [6, 34, 35, 36].

This work has limitations linked to the lack of implementation of these tools due to the investment that the centers must make in technical infrastructure for their use (virtual reality glasses). However, the various studies that have been developing this methodology in recent years mean that this technology may be implemented in education centers in the future. This is considered a strength of the work and opens the possibility of new lines of research.

## 5 CONCLUSION

Based on the results explained and in relation to the previously stated objectives, two fundamental conclusions are reached. On the one hand, the importance of establishing a good curricular context for the subject to be studied. On the other hand, the importance of choosing an appropriate methodology. Firstly, the update of curricular concepts on the maturation process of foot bones based on existing scientific literature is provided. Secondly, unprecedented didactic tools are developed, where the maturation processes of the bones forming the foot skeleton are digitally created. The implementation of virtual models in the podiatry curriculum can facilitate the understanding of the ossification process, enhancing the quality of education and future clinical practice.

## AUTHOR CONTRIBUTIONS

**Ana Cristina Moreno‐Marin**: Conceptualization; investigation; writing—original draft; writing—review & editing. **Manuel Pardo Rios**: Funding acquisition; project administration; data curation; formal analysis; writing—review & editing. **Eva Lopezosa‐Reca**: Methodology; visualization; writing—review & editing. **Cristina Molina García**: Resources; software; writing—review & editing. **Salvador Díaz‐Miguel**: Supervision; validation; writing—review & editing. **Beatriz Gómez‐Martín**: Conceptualization; investigation; writing—review & editing. **Paula Cobos‐Moreno**: Investigation; writing—review & editing.

## CONFLICT OF INTEREST STATEMENT

The authors declare that they have no competing interests.

## ETHICS STATEMENT

Not applicable.

## CONSENT FOR PUBLICATION

Not applicable.

## INSTITUTIONAL REVIEW BOARD STATEMENT

Not applicable.

## INFORMED CONSENT STATEMENT

Not applicable.

## Data Availability

Data sharing is not applicable to this article as no new data were created or analyzed in this study.
